# Factors associated with persistent bacteraemia among patients with suspected infective endocarditis

**DOI:** 10.1007/s15010-025-02537-5

**Published:** 2025-04-07

**Authors:** André Teixeira-Antunes, Virgile Zimmermann, Nicolas Fourré, Nicoleta Ianculescu, Pierre Monney, Georgios Tzimas, Laurence Senn, Piergiorgio Tozzi, Matthias Kirsch, Benoit Guery, Matthaios Papadimitriou-Olivgeris

**Affiliations:** 1 Infectious Diseases Service, Lausanne University Hospital and University of Lausanne, Lausanne, Switzerland; 2 Department of Cardiology, Lausanne University Hospital and University of Lausanne, Lausanne, Switzerland; 3 Infection Prevention and Control Unit, Lausanne University Hospital and University of Lausanne, Lausanne, Switzerland; 4 Department of Cardiac Surgery, Lausanne University Hospital and University of Lausanne, Lausanne, Switzerland; 5 Infectious Diseases Service, Hospital of Valais and Institut Central des Hôpitaux, Sion, 1951 Switzerland

**Keywords:** Persistent bacteraemia, Infective endocarditis, Follow-up blood cultures, Bone and joint infection, Sepsis

## Abstract

**Purpose:**

To ascertain the predictors of persistent bacteraemia among patients with suspected infective endocarditis (IE) and those with IE.

**Methods:**

Retrospective study.

**Setting:**

This study conducted at a Swiss university hospital (2015–2023) included adult patients with bacteraemia and suspected IE. Persistent bacteraemia was defined as continued positive blood cultures with the same microorganism for at least 48 h from antibiotic treatment initiation. Endocarditis Team classified cases as IE or not IE.

**Results:**

Among 2312 episodes of suspected IE, *S. aureus* was the most common pathogen (1045 episodes; 45%). IE (644; 28%) was the most prevalent infection type. Persistent bacteraemia was observed in 480 (21%) episodes and was independently associated with *S. aureus*, ≥ 2 positive sets of index blood cultures, resistant bacterium, sepsis, IE, central venous catheter-associated bacteraemia, and acute native bone and joint infections (BJIs), while, streptococcal bacteraemia, appropriate initial antimicrobial treatment and, performance of source control interventions within 48 h were associated with rapid blood culture clearance. Of the 644 IE episodes, persistent bacteraemia was observed in 196 (30%) and was associated with obesity, *S. aureus*, ≥ 2 positive sets of index blood cultures, resistant bacterium, acute native BJIs, immunologic phenomena, thoracic embolic events, while streptococcal bacteraemia and performance of source control interventions within 48 h were associated with rapid clearance of blood cultures.

**Conclusions:**

Persistent bacteraemia was associated with *S. aureus* and BJI. Delaying source control interventions may increase the risk of persistent bacteraemia. No specific intracardiac lesion was associated with persistent bacteraemia in IE episodes.

**Supplementary Information:**

The online version contains supplementary material available at 10.1007/s15010-025-02537-5.

## Introduction

Despite significant advances in microbiology, blood cultures remain the cornerstone of diagnosing infective endocarditis (IE) [1], identifying the microbial cause in over 90% of cases [1]. Their importance is emphasized by their inclusion as a major Duke criterion, alongside the detection of typical intracardiac lesions [1, 2]. When initial assessments yield inconclusive results, positive follow-up blood cultures raise the suspicion of IE [3], prompting clinicians to pursue further diagnostics, including echocardiography [4–7], or thoracoabdominal and cerebral imaging to detect embolic events or metastatic complications, including bone and joint infections (BJIs) [6–8].

In clinical practice, follow-up blood cultures are part of the management of intravascular infections like IE, *Staphylococcus aureus* bacteraemia, candidemia, and in cases of persistent of fever despite appropriate antimicrobial therapy [1, 2, 9, 10]. They play a dual role in guiding diagnostic and therapeutic decisions and serve as a prognostic marker. Indeed, persistent bacteraemia for at least 48 h signals an ongoing infection, often due to inadequate antimicrobial therapy or insufficient source control [11–16], both associated with worse outcomes [17–19]. Specifically, persistently positive blood cultures beyond 7 days despite appropriate antibiotic therapy and adequate control of metastatic foci are considered an indication for urgent valve surgery [1, 2]. However, this cut-off is arbitrary, and no study to date has evaluated predictors of persistent bacteraemia lasting at least seven days among patients with valve-associated IE.

The relationship between persistent bacteraemia and IEhas been extensively investigated [3, 11–16], but data on factors associated with persistent bacteraemia specifically in IE patients remain limited [14, 20]. Furthermore, in most patients with suspected IE, the diagnosis is confirmed or ruled out after 48 h [21], and follow-up blood cultures are typically performed at this time while awaiting the final diagnosis. Therefore, this study aimed to identify factors associated with persistent bacteraemia in patients with suspected or confirmed IE.

## Methods

### Study design

This study was conducted at the Lausanne University Hospital in Switzerland, merging two cohorts: (1) the retrospective bacteraemia cohort from January 2015 to December 2021, and (2) the prospective cohort of patients suspected of IE from January 2022 to December 2023. Ethical approval for the study was obtained from the ethics committee of the Canton of Vaud (CER-VD 2017–02137, CER-VD 2021–02516).

### Patients

Inclusion criteria were adult patients (≥ 18 years old) with bacteraemia and clinical suspicion of IE, defined as echocardiography performed specifically for the search of IE, and for the retrospective cohort the absence of written refusal to use their data and for the prospective cohort, the presence of written consent. Exclusion criteria were the absence of follow-up blood cultures, and death within 48 h from antibiotic treatment initiation without negative follow-up blood cultures.

Episodes in the bacteraemia cohort were identified through the microbiology database, while those in the suspected IE cohort were prospectively identified in the echocardiography service. Data regarding demographics, comorbidities, cardiac predisposing factors, setting of infection onset, blood culture data, systemic manifestations, type of infection, embolic events, immunological phenomena, cardiac lesions, antimicrobial treatment and source control were retrieved from patients’ electronic health records by internal medicine and infectious diseases consultants. All data were verified by an infectious diseases consultant.

### Management of cases of suspected IE

For suspected IE cases follow-up blood cultures were taken every 24–48 h until clearance and transthoracic echocardiography (TTE) was recommended. The decision to perform additional cardiac imaging studies like transoesophageal echocardiography (TOE), ^18^F-Fluorodeoxyglucose Positron Emission Tomography/Computed Tomography (^18^F-FDG PET/CT) or cardiac CT was made by the infectious diseases consultant or the Endocarditis Team. Patients with bacteraemia with suspicion of IE were initially treated as IE-specific antibiotic regimes until diagnosis was confirmed or excluded [2].

### Definitions

Persistent bacteraemia was defined as having at least one positive blood cultures by the same bacterium at 48 h or later after the antibiotic treatment initiation, as proposed in previous study on *S. aureus* [22]. For the subanalysis evaluating the surgical indication for persistently positive blood cultures, a threshold of 168 h was used to define persistent bacteraemia. Sepsis and septic shock were defined as per the Sepsis-3 International Consensus [23]. Obesity was defined as a body mass index ≥ 30 kg/m^2^. Antimicrobial treatment initiation was defined as the hour of administration of the first antimicrobial. Since 2018, IE diagnosis was made by the Endocarditis Team; before 2018, two expert clinicians (MPO, PM), who were part of the Endocarditis Team since 2018, served the role of adjudicating cases. IE cases were classified as definite, possible, or rejected IE based on the 2023 ISCVID Duke criteria [24]. The determination of other foci of infection was based on the assessment by the ID consultant, taking into account clinical, radiological, microbiological, and operative findings. Source control procedures included the removal of venous catheters, surgical or imaging-guided drainage of infected collections, joint fluid drainage, correction of urinary-tract obstruction, removal of cardiac implantable electronic devices (CIED), and cardiac surgery for heart failure indications [1]. The initial antimicrobial regimen was considered appropriate if the infecting isolate was susceptible to at least one administered antimicrobial. Resistant bacteria included methicillin-resistant *S. aureus* (MRSA) or coagulase negative staphylococci, penicillin-resistant streptococci, and amoxicillin-resistant enterococci.

### Statistical analysis

Data analysis was conducted using SPSS version 26.0 (SPSS, Chicago, Illinois, USA). Categorical variables were analysed using the *chi*2 or Fisher exact test and continuous variables with Mann-Whitney *U* test. Bivariable and multivariable logistic regression analyses were performed with dependent variable being presence or absence of persistent bacteraemia in patients with suspected IE and those with IE. Variables with *P <* 0.1 that did not contribute to multicollinearity (variance inflation factor assessment) were used in multivariable analyses. Adjusted odds ratios (aORs) and 95% confidence intervals (CIs) were calculated to evaluate the strength of any association. All statistic tests were 2-tailed, and *P* < 0.05 was considered statistically significant.

## Results

### Study population

**Fig. 1:**
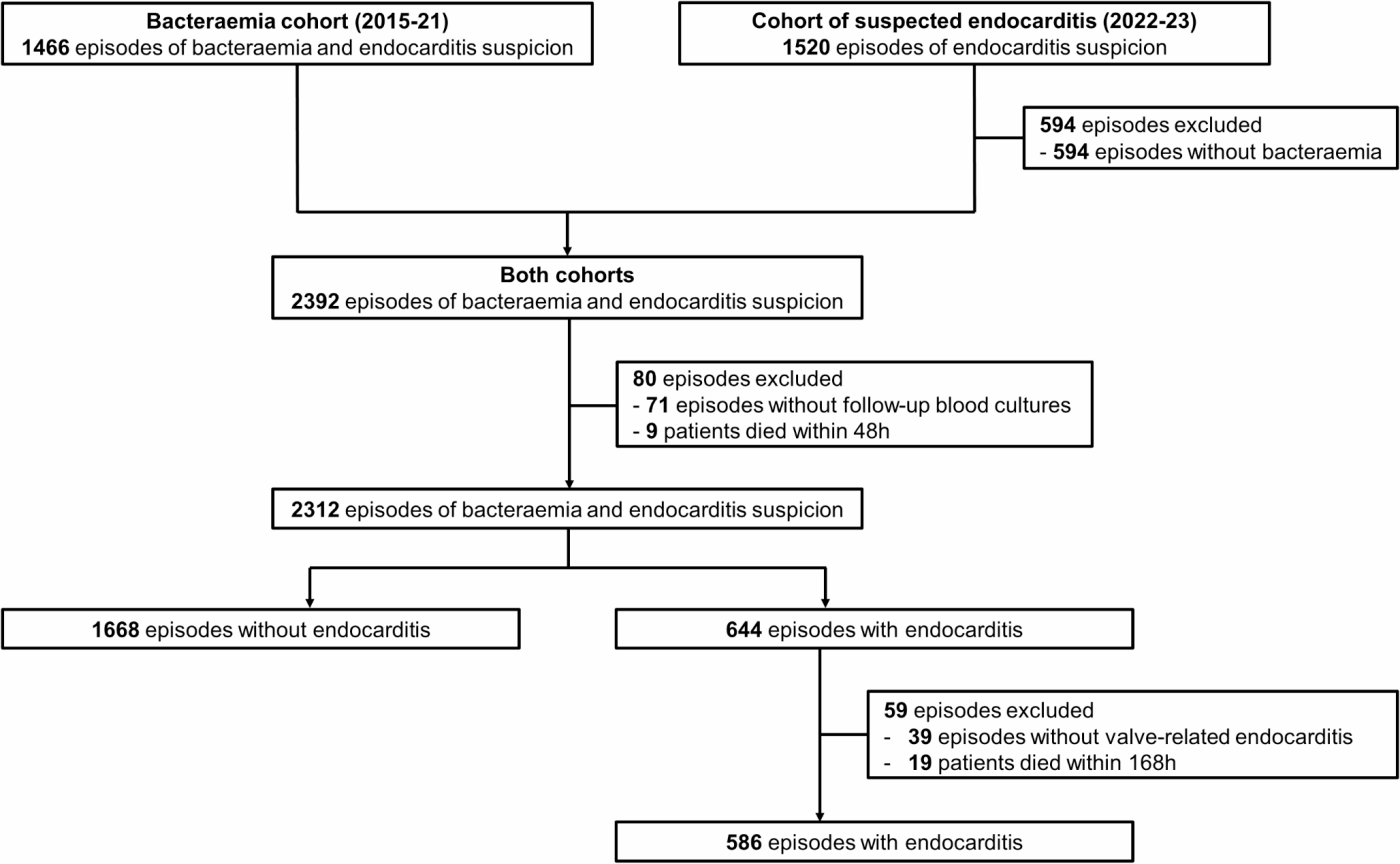
Flowchart of patients

Of the 2986 episodes across both cohorts, 2312 were included (Fig. 1). Episodes were excluded based on specific criteria: 594 had negative index blood cultures, 71 lacked follow-up blood cultures, and 9 patients succumbed within 48 h. All patients had the first follow-up blood culture within 48 h from the antimicrobial treatment initiation.

The most frequently isolated pathogen was *S. aureus* (1045; 45%), followed by streptococci (511; 22%), and enterococci (412; 18%). Infections caused by resistant bacteria were noted in 367 (16%) episodes, comprising 70/1045 (7%) MRSA, 125/176 (71%) methicillin-resistant coagulase-negative staphylococci, 49/511 (10%) penicillin-resistant streptococci, and 129/412 (31%) amoxicillin-resistant enterococci; three episodes having a bacteraemia with two resistant bacteria.

IE (644; 28%) was the most prevalent infection type, followed by BJI (526; 23%), and central venous catheter-associated bacteraemia (293; 13%). Among the 644 IE episodes, 490 (76%) were classified as definite IE and 154 (24%) as possible IE according to the 2023 ISCVID Duke criteria. Sepsis was observed in 1002 (43%) episodes. Cardiac imaging procedures like TTE, TOE, ^18^F-FDG PET/CT and cardiac-CT were performed in 2161 (94%), 1054 (46%), 366 (16%), and 59 (3%) episodes, respectively.

Persistent bacteraemia was observed in 480 (21%) episodes. Among episodes with bacteraemia by either *S. aureus*, streptococci, or enterococci, persistent bacteraemia was present in 32% (336/1045), 3% (13/511), 16% (65/412), respectively. Table 1 shows the characteristics of suspected IE episodes with and without persistent bacteraemia. IE was more prevalent among episodes with persistent bacteraemia compared to those without (44% *versus* 27%; *P* < 0.001). Multivariable analysis (Table 2) demonstrated various factors significantly associated with persistent bacteraemia, such as *S. aureus* bacteraemia (aOR 2.81, 95% CI 2.04–3.87), two or more positive sets of index blood cultures (3.08, 2.13–4.44), bacteraemia by a resistant bacterium (2.04, 1.47–2.83), sepsis (1.48, 1.18–1.86), IE (2.44, 1.85–3.22), central venous catheter-associated bacteraemia (1.82, 1.26–2.65), and BJI (1.61, 1.20–2.15). Conversely, streptococcal bacteraemia (0.12, 0.07–0.23), appropriate initial antimicrobial treatment (0.58, 0.41–0.82) and, when indicated, performance of source control interventions within 48 h (0.39, 0.29–0.52) were associated with rapid blood culture clearance.

**Table 1:** Characteristics of suspected infective endocarditis episodes with and without persistent bacteraemia

	No persistent bacteraemia (*n* = 1832)	Persistent bacteraemia (*n* = 480)	*P*
Demographics			
Male sex	1297 (71%)	335 (70%)	0.694
Age (years)	69 (57–79)	67 (56–77)	0.065
Age > 60 years	1253 (68%)	325 (68%)	0.783
Co-morbidities			
Malignancy (solid organ or hematologic)	458 (25%)	100 (21%)	0.063
Diabetes mellitus	482 (26%)	146 (30%)	0.074
Chronic kidney disease (moderate or severe)	442 (24%)	112 (23%)	0.764
Chronic obstructive pulmonary disease	235 (13%)	55 (12%)	0.440
Congestive heart failure	187 (10%)	49 (10%)	1.000
Cirrhosis	145 (8%)	45 (9%)	0.305
Obesity (body mass index ≥ 30 kg/m²)	405 (22%)	116 (24%)	0.357
Immunosuppression	339 (19%)	85 (18%)	0.741
Charlson Comorbidity Index	5 (3–7)	4 (2–7)	0.146
Setting of infection onset			
Community	826 (45%)	248 (52%)	0.012
Healthcare-associated	335 (18%)	89 (19%)	
Nosocomial	671 (37%)	143 (30%)	
Isolated pathogen			
*S. aureus*	709 (39%)	336 (70%)	< 0.001
Coagulase negative staphylococci	140 (8%)	36 (10%)	0.186
Streptococci	498 (27%)	13 (3%)	< 0.001
Enterococci	347 (19%)	65 (14%)	0.006
Other Gram-positive	61 (3%)	10 (2%)	0.736
HACEK	23 (1%)	0 (0%)	0.008
Other Gram-negative	235 (13%)	47 (10%)	0.072
Polymicrobial bacteraemia	205 (11%)	48 (10%)	0.511
>1 positive set of index blood cultures	1347 (74%)	440 (92%)	< 0.001
Resistant bacteria^a^	275 (15%)	95 (20%)	0.014
Manifestations			
Fever	1528 (83%)	399 (83%)	0.891
Sepsis	741 (40%)	261 (54%)	< 0.001
Septic shock	237 (13%)	98 (20%)	< 0.001
Type of infection			
Infective endocarditis	448 (25%)	196 (41%)	< 0.001
Bacteraemia of unknown origin	174 (10%)	19 (4%)	< 0.001
Central venous catheter-associated	217 (12%)	76 (16%)	0.025
Peripheral venous catheter -associated	122 (7%)	21 (4%)	0.070
Skin and soft tissue infection	209 (11%)	35 (7%)	0.009
Bone and joint infection	360 (20%)	166 (35%)	< 0.001
Acute native bone and joint infection	186 (10%)	133 (28%)	< 0.001
Septic arthritis	106 (6%)	75 (16%)	< 0.001
Vertebral and non-vertebral osteomyelitis	87 (5%)	80 (17%)	< 0.001
Orthopedic implant-associated infection	112 (6%)	42 (9%)	0.050
Low-respiratory tract infection	117 (6%)	17 (4%)	0.016
Urinary-tract infection	89 (5%)	6 (1%)	< 0.001
Intraabdominal infection	164 (9%)	33 (7%)	0.168
Other type of infection	153 (8%)	48 (10%)	0.274
Appropriate initial antimicrobial treatment	1651 (90%)	407 (85%)	0.001
Source control			
Not warranted	880 (48%)	160 (33%)	
Warranted; performed within 48 h	592 (32%)	119 (25%)	
Warranted; not performed within 48 h	360 (20%)	201 (42%)	< 0.001

Data are depicted as number (percentage) or median (Q1-Q3)

^a^included methicillin resistant staphylococci, penicillin resistant streptococci, and amoxicillin resistant enterococci

**Table 2:** Multivariable analysis of persistent bacteraemia among patients with suspected infective endocarditis

	*P*	aOR (95% CI)
Age	0.054	0.99 (0.99-1.00)
Malignancy (solid organ or hematologic)	0.299	1.17 (0.87–1.56)
Diabetes mellitus	0.287	1.15 (0.89–1.49)
Community-acquired	0.299	1.17 (0.99–1.68)
*S. aureus*	< 0.001	2.81 (2.04–3.87)
Streptococci	< 0.001	0.12 (0.07–0.23)
Enterococci	0.883	0.97 (0.67–1.41)
> 1 positive set of index blood cultures	< 0.001	3.08 (2.13–4.44)
Resistant bacteria^a^	< 0.001	2.04 (1.47–2.83)
Sepsis	0.001	1.48 (1.18–1.86)
Infective endocarditis	< 0.001	2.44 (1.85–3.22)
Central venous catheter-associated bacteraemia	< 0.001	1.82 (1.26–2.65)
Acute native bone and joint infection	0.001	1.61 (1.20–2.15)
Appropriate initial antimicrobial treatment	0.002	0.58 (0.41–0.82)
Source control		
Warranted; not performed within 48 h	reference	reference
Warranted; performed within 48 h	< 0.001	0.39 (0.29–0.52)
Not warranted	< 0.001	0.48 (0.35–0.64)

aOR: adjusted odds ratios, CI: confidence interval

^a^included methicillin resistant staphylococci, penicillin resistant streptococci, and amoxicillin resistant enterococci

Of the 644 IE episodes, persistent bacteraemia was observed in 196 (30%). Among episodes with IE by either *S. aureus*, streptococci, or enterococci, persistent bacteraemia was present in 56% (153/272), 3% (6/175), 13% (14/110), respectively. Table 3 illustrates the characteristics of IE episodes with and without persistent bacteraemia. Obesity (aOR 1. 73, 95% CI 1.03–2.93), *S. aureus* bacteraemia (3.72, 2.63–6.82), two or more positive sets of index blood cultures (3.28, 1.06–10.15), bacteraemia by a resistant bacterium (3.20, 1.60–6.43), acute native bone and joint infection (2.86, 1.66–4.92), immunologic phenomena (2.84, 1.24–6.55), thoracic embolic events (2.35, 1.07–5.16) were independently associated with persistent bacteraemia, while bacteraemia by streptococci (0.11, 0.04–0.28), and, when indicated, performance of source control interventions within 48 h (0.38, 0.18–0.78) were associated with rapid clearance of blood cultures (Table 4).

**Table 3:** Characteristics of infective endocarditis episodes with and without persistent bacteraemia

	No persistent bacteraemia (*n* = 448)	Persistent bacteraemia (*n* = 196)	*P*
Demographics			
Male sex	332 (74%)	138 (70%)	0.336
Age (years)	69 (57–80)	67 (53–76)	0.037
Age > 60 years	317 (71%)	125 (64%)	0.080
Co-morbidities			
Malignancy (solid organ or hematologic)	48 (11%)	22 (11%)	0.891
Diabetes mellitus	100 (22%)	54 (28%)	0.161
Chronic kidney disease (moderate or severe)	83 (19%)	50 (26%)	0.057
Chronic obstructive pulmonary disease	48 (11%)	27 (14%)	0.286
Congestive heart failure	48 (11%)	21 (11%)	1.000
Cirrhosis	26 (6%)	18 (9%)	0.128
Obesity (body mass index ≥ 30 kg/m²)	80 (18%)	56 (29%)	0.003
Immunosuppression	25 (6%)	19 (10%)	0.063
Charlson Comorbidity Index	4 (2–7)	4 (2–7)	0.999
Cardiac predisposing factors			
Intravenous drug use	28 (6%)	25 (13%)	0.008
Prior infective endocarditis	42 (9%)	21 (11%)	0.666
Prosthetic valve	153 (34%)	48 (25%)	0.016
CIED	77 (17%)	42 (21%)	0.225
Setting of infection onset			
Community	330 (74%)	136 (69%)	0.464
Healthcare-associated	62 (14%)	33 (17%)	
Nosocomial	56 (13%)	27 (14%)	
Isolated pathogen			
*S. aureus*	119 (27%)	153 (78%)	< 0.001
Coagulase negative staphylococci	29 (7%)	20 (10%)	0.108
Streptococci	169 (38%)	6 (3%)	< 0.001
Enterococci	96 (21%)	14 (7%)	< 0.001
Other Gram-positive	14 (3%)	3 (2%)	0.297
HACEK	19 (4%)	0 (0%)	0.001
Other Gram-negative	17 (4%)	6 (3%)	0.818
Polymicrobial bacteraemia	15 (3%)	6 (3%)	1.000
>1 positive set of index blood cultures	415 (93%)	191 (97%)	0.017
Resistant bacteria^a^	38 (9%)	30 (15%)	0.012
Manifestations			
Fever	374 (84%)	166 (85%)	0.729
Sepsis	181 (40%)	118 (56%)	< 0.001
Septic shock	64 (14%)	55 (28%)	< 0.001
Embolic events before antimicrobial treatment	177 (40%)	97 (50%)	0.020
Cerebral	110 (25%)	46 (24%)	0.842
Non-cerebral			
Limbs	16 (4%)	16 (8%)	0.018
Ocular	47 (11%)	29 (15%)	0.144
Thoracic	20 (5%)	34 (17%)	< 0.001
Abdominal	69 (15%)	43 (22%)	0.054
Immunologic phenomena	23 (5%)	21 (11%)	0.016
Bone and joint infection	60 (13%)	69 (35%)	< 0.001
Acute native bone and joint infection	54 (12%)	63 (32%)	< 0.001
Septic arthritis	27 (6%)	41 (21%)	< 0.001
Vertebral and non-vertebral osteomyelitis	28 (6%)	32 (16%)	< 0.001
Orthopedic implant-associated infection	10 (2%)	9 (5%)	0.128
Site of infection			
Aortic valve	238 (53%)	95 (49%)	0.304
Mitral valve	197 (44%)	68 (35%)	0.030
Tricuspid valve	27 (6%)	29 (15%)	< 0.001
Pulmonary valve	8 (2%)	4 (2%)	0.762
Multivalvular	46 (10%)	17 (9%)	0.568
CIED-lead	36 (8%)	34 (17%)	0.001
Other intracardial site of infection	2 (0.4%)	1 (0.5%)	1.000
Type of valve			
Native	296 (66%)	138 (80%)	0.274
Prosthetic	132 (30%)	45 (23%)	0.103
Intracardiac lesions			
Vegetation	276 (62%)	124 (63%)	0.724
Vegetation ≥ 10 mm	163 (36%)	82 (42%)	0.217
Abscess	88 (20%)	35 (18%)	0.663
Other lesions^c^	76 (17%)	24 (12%)	0.156
Appropriate initial antimicrobial treatment	409 (91%)	178 (91%)	0.880
Source control			
Not warranted	313 (70%)	96 (49%)	
Warranted; performed within 48 h	53 (12%)	23 (12%)	
Warranted; not performed within 48 h	82 (18%)	77 (39%)	0.011

Data are depicted as number (percentage) or median (Q1-Q3), CIED: cardiac implantable electronic devices

^a^included methicillin resistant staphylococci, penicillin resistant streptococci, and amoxicillin resistant enterococci

**Table 4:** Multivariable analysis of predictors of persistent bacteraemia among patients with infective endocarditis

	*P*	aOR (95% CI)
Age > 60 years	0.407	0.79 (0.45–1.38)
Chronic kidney disease (moderate or severe)	0.464	1.22 (0.72–2.06)
Obesity (body mass index ≥ 30 kg/m²)	0.039	1.73 (1.03–2.93)
Immunosuppression	0.213	1.67 (0.75–3.69)
Intravenous drug use	0.855	1.08 (0.47–2.51)
*S. aureus*	< 0.001	3.72 (2.63–6.82)
Streptococci	< 0.001	0.11 (0.04–0.28)
Enterococci	0.109	0.55 (0.26–1.15)
> 1 positive set of index blood cultures	0.040	3.28 (1.06–10.15)
Resistant bacteria^a^	0.001	3.20 (1.60–6.43)
Sepsis	0.330	1.25 (0.80–1.97)
Acute native bone and joint infection	< 0.001	2.86 (1.66–4.92)
Thoracic embolic events	0.034	2.35 (1.07–5.16)
Immunologic phenomena	0.014	2.84 (1.24–6.55)
CIED-lead infective endocarditis	0.462	1.31 (0.64–2.68)
Tricuspid valve infective endocarditis	0.792	1.12 (0.50–2.50)
Source control		
Warranted; not performed within 48 h	reference	reference
Warranted; performed within 48 h	0.009	0.38 (0.18–0.78)
Not warranted	0.122	0.64 (0.36–1.13)

aOR: adjusted odds ratios, CI: confidence interval, CIED: cardiac implantable electronic devices

^a^included methicillin resistant staphylococci, penicillin resistant streptococci, and amoxicillin resistant enterococci

For the evaluation of the surgical indication of persistently positive blood cultures for at least 7 days despite appropriate antibiotic therapy and adequate control of metastatic foci, 586 episodes of valve IE in patients who survived beyond seven days were included (Fig. 1). Persistent bacteraemia for at least 7 days was observed in 56 (10%) episodes (Supplementary Table 1). Of them, only 4/56 (7%) episodes met the conditions of the aforementioned indication. *S. aureus* bacteraemia (aOR 10.24, 95% CI 3.80-27.61), bacteraemia by a resistant bacterium (3.20, 1.60–6.43), and acute native bone and joint infection (3.02, 1.38–6.62), were independently associated with persistent bacteraemia, while performance of source control interventions within 168 h, when indicated, (0.25, 0.01–0.79) or absence of indication for source control interventions (0.04, 0.01–0.10) were associated with rapid clearance of blood cultures (Supplementary Table 2).

## Discussion

This study, aimed at characterizing episodes of persistent bacteraemia in patients with suspected IE and those with IE, found that the prevalence of persistent bacteraemia was 21% and 30%, respectively, and was more common in episodes involving *S. aureus* bacteraemia and acute native BJI.

The most notable observation was the strong association of *S. aureus* with persistent bacteraemia (32%), in contrast to lower rates in enterococcal (16%) and particularly streptococcal bacteraemia (3%). This trend persisted in IE episodes. Specifically, among patients with non-staphylococcal IE, only 7% exhibited persistent bacteraemia, consistent with prior reports 4%) [20]. Another study similarly linked *S. aureus* to persistent bacteraemia, whereas streptococcal bacteraemia was associated with a higher likelihood of clearance [14]. Guidelines recommend collecting follow-up blood cultures within 48–72 h to assess treatment effectiveness in IE patients; [1] however, our findings challenge this approach for patients with non-staphylococcal IE. Interestingly, our results differ from a previous study that reported no significant difference in persistent bacteraemia rates between enterococcal IE (13%) and IE caused by other pathogens (11%). Possible reasons for this discrepancy include the higher proportion of culture-negative cases (10%) in non-enterococcal IE, the absence of data on follow-up blood cultures, and the lower prevalence of *S. aureus* in that cohort (21%), which is half the proportion observed in our study [25].

Persistent bacteraemia was also associated to the number of positive sets in the initial blood culture collection, previously linked with an increased likelihood of IE [16]. Multiple positive blood cultures strongly indicated IE in cases of streptococcal or enterococcal bacteraemia, forming the basis of prediction tools such as NOVA, DENOVA, and HANDOC [26–29].

Our findings align with studies demonstrating an association between persistent bacteraemia and metastatic infections in general [11, 12, 15, 30], particularly in cases of IE [13, 30, 31] and acute native BJIs [11, 30]. Central venous catheter-associated bacteraemia was also linked to persistent bacteraemia, reinforcing evidence of the role of prosthetic material infections, such as intravascular catheters, in the persistence of bacteraemia [11–14]. These findings emphasize the critical importance of prompt source control, as identified in both previous and current studies, in achieving rapid clearance of blood cultures [15, 16]. While persistent bacteraemia is often associated with adverse outcomes, such as prolonged hospitalization and increased mortality [11, 12, 22, 31], our findings, in line with prior research [11–16], suggest that persistent bacteraemia itself might not be the primary cause of these outcomes. Instead, it likely serves as a marker of inadequate source control, a pivotal factor influencing mortality rates [17–19]. Consequently, positive follow-up blood cultures necessitate thorough diagnostic reassessment, evaluation of source control measures, and potential modifications to antimicrobial therapy [6, 7].

Bacteraemia caused by resistant pathogens was associated with persistent bacteraemia across the entire cohort and among IE cases, even when the appropriateness of initial antimicrobial treatment was comparable between IE patients with and without persistent bacteraemia. The likely stems from reliance on vancomycin as the therapeutic option for resistant bacteria, known to be less effective than beta-lactams [32]. Similarly, previous studies have linked MRSA infections to persistent bacteraemia [11, 13].

Among IE cases, obesity was associated with persistent bacteraemia, potentially due to altered pharmacokinetics and pharmacodynamics affecting antimicrobial dosing in obese individuals [33]. In fact, in our center, contrary to renal function, site and severity of infection, body weight did not influence the dosing of beta-lactams which probably led to inadequate dosing in some patients.

Although persistent bacteraemia has been studied in relation to IE among bacteraemic patients [11–16], to the best of our knowledge, this is the first study to specifically investigate risk factors for persistent bacteraemia in IE patients. Notably, no intracardiac lesion, such as large vegetations, abscesses, or other paravalvular complications, was associated with persistent bacteraemia. Instead, extracardiac complications, such as metastatic infections (septic arthritis, spondylodiscitis), that usually warrant source control interventions, and thoracic embolic events were independently linked to persistent bacteraemia. The latter were common in patients with CIED-lead IE or intravenous drug use-associated tricuspid valve IE, but did not emerge as independent predictors in multivariable analysis.

While 10% of episodes with valve-associated IE had persistent bacteraemia for at least 7 days, less than 1% met the criteria for surgical indication due to persistently positive blood cultures [1, 2]. The majority of episodes with persistent positive blood cultures for at least seven days were attributable to inadequate control of metastatic foci.

Our study has several limitations. First, it was conducted at a single center in a setting with a low prevalence of multidrug-resistant pathogens, potentially limiting the generalizability of our findings. Second, a significant proportion of patients were included retrospectively. Third, the interval for follow-up blood cultures varied (every 1–2 days until clearance), and in some episodes, follow-up blood cultures may not have been taken precisely at the 48-hour mark. Since we did not systematically document the exact timing of each subsequent blood culture, this could have led to missed cases of persistent bacteraemia. However, after excluding the 1440 episodes without a positive follow-up blood culture and the 480 episodes with positive blood cultures at 48 h or later, only 392 patients remained where misclassification might have occurred. Fourth, in cases where IE diagnosis was not definitive but highly suspected, the persistence of positive blood cultures may have influenced the Endocarditis Team to classify those episodes as IE.

In summary, persistent bacteraemia is a common finding in patients with suspected IE and is associated with conditions such as acute native BJIs. Our findings highlight the importance of prompt source control, as delays in achieving source control beyond 48 h may increase the risk of persistent bacteraemia. In patients with IE, persistent bacteraemia should prompt clinicians to investigate metastatic complications, such as septic arthritis, spondylodiscitis, or thoracic embolic events. Interestingly, no specific intracardiac lesion was associated with persistent bacteraemia. Persistent bacteraemia was notably more frequent in patients with *S. aureus* bacteraemia, a condition where follow-up blood cultures are already standard practice. Conversely, in patients with streptococcal IE, persistent bacteraemia was uncommon; thus, routine follow-up blood cultures in streptococcal IE may warrant reevaluation. Among patients with valve IE, the primary cause of persistent positive blood cultures lasting at least seven days was inadequate source control of metastatic foci, with only a small minority meeting the criteria for surgical indication due to persistently positive blood cultures.

## Electronic supplementary material

Below is the link to the electronic supplementary material.

Supplementary Material 1

## Data Availability

The data that support the findings of this study are available from the corresponding author upon reasonable request.
