# Supplementary material for: Factors associated with persistent bacteraemia among patients with suspected infective endocarditis
Source: Infection. 2025 Apr 7;53(5):2015–24. doi: 10.1007/s15010-025-02537-5 (PMC12460498; doi:10.1007/s15010-025-02537-5)
Supplement: Supplementary file 1 — Supplementary Material 1 [file 15010_2025_2537_MOESM1_ESM.pdf]

Supplementary Table 1. Characteristics of valve-associated infective endocarditis episodes with and without persistent bacteraemia for at least 168 hours

	**No persistent bacteraemia (n=530)**	**Persistent bacteraemia (n=56)**	***P***
Demographics			
Male sex	392 (74%)	40 (71%)	0.750
Age (years)	69 (55-78)	62 (53-73)	0.056
Age >60 years	365 (69%)	29 (52%)	0.016
Co-morbidities			
Malignancy (solid organ or haematologic)	59 (11%)	6 (11%)	1.000
Diabetes mellitus	128 (24%)	13 (23%)	1.000
Chronic kidney disease (moderate or severe)	103 (19%)	15 (27%)	0.219
Chronic obstructive pulmonary disease	61 (12%)	6 (11%)	1.000
Congestive heart failure	49 (9%)	4 (7%)	0.807
Cirrhosis	32 (6%)	9 (16%)	0.011
Obesity (body mass index ≥30 kg/m²)	103 (19%)	19 (34%)	0.015
Immunosuppression	34 (6%)	4 (7%)	0.776
Charlson Comorbidity Index	4 (2-7)	4 (2-7)	0.718
Cardiac predisposing factors			

Intravenous drug use	44 (8%)	6 (11%)	0.461
Prior infective endocarditis	57 (11%)	4 (7%)	0.496
Prosthetic valve	180 (34%)	6 (11%)	<0.001
CIED	72 (14%)	6 (11%)	0.681
Setting of infection onset			
Community	388 (73%)	42 (75%)	0.874
Healthcare-associated	76 (14%)	11 (20%)	
Nosocomial	66 (13%)	3 (5%)	
Isolated pathogen			
S. aureus	188 (36%)	47 (84%)	<0.001
Coagulase negative staphylococci	37 (7%)	7 (13%)	0.175
Streptococci	170 (32%)	1 (2%)	<0.001
Enterococci	101 (19%)	1 (2%)	<0.001
Other Gram-positive	15 (3%)	0 (0%)	0.382
HACEK	19 (4%)	0 (0%)	0.242
Other Gram-negative	20 (4%)	1 (2%)	0.710
Polymicrobial bacteraemia	20 (4%)	1 (2%)	0.710
>1 positive set of index blood cultures	497 (94%)	55 (98%)	0.237
Resistant bacteria[a]	51 (10%)	13 (23%)	0.005

Manifestations			
Fever	447 (84%)	44 (79%)	0.256
Sepsis	226 (43%)	34 (61%)	0.011
Septic shock	20 (4%)	1 (2%)	0.710
Embolic events within168 hours	291 (55%)	36 (64%)	0.204
Cerebral	193 (36%)	20 (36%)	1.000
Non-cerebral			
Limbs	71 (13%)	9 (316%)	0.543
Ocular	28 (5%)	6 (11%)	0.124
Thoracic	37 (7%)	20 (36%)	<0.001
Abdominal	127 (24%)	16 (29%)	0.513
Immunologic phenomena	35 (7%)	8 (14%)	0.053
Bone and joint infection	93 (18%)	28 (50%)	<0.001
Acute native bone and joint infection			
Septic arthritis	47 (9%)	17 (30%)	<0.001
Vertebral and non-vertebral osteomyelitis	42 (8%)	15 (27%)	<0.001
Orthopedic implant-associated infection	16 (3%)	1 (2%)	1.000
Site of infection			
Aortic valve	296 (56%)	28 (50%)	0.480

Mitral valve	236 (45%)	18 (32%)	0.089
Tricuspid valve	41 (8%)	13 (23%)	0.001
Pulmonary valve	12 (2%)	0 (0%)	0617
Multivalvular	57 (11%)	4 (7%)	0.496
CIED-lead	24 (5%)	5 (9%)	0.183
Other intracardial site of infection	2 (0.4%)	1 (2%)	0.261
Type of valve			
Native	373 (70%)	49 (88%)	0.007
Prosthetic	162 (31%)	7 (13%)	0.005
Intracardiac lesions			
Vegetation	337 (64%)	36 (64%)	1.000
Vegetation ≥10mm	205 (39%)	21 (38%)	0.886
Abscess	114 (22%)	8 (14%)	0.230
Other lesions[c]	96 (18%)	4 (7%)	0.039
Appropriate initial antimicrobial treatment within 48 hours	501 (95%)	53 (95%)	1.000
Source control			
Not warranted	375 (71%)	19 (34%)	
Warranted; performed within 168 hours	143 (27%)	11 (20%)	
Warranted; not performed within 168 hours	12 (2%)	26 (46%)	<0.001

Valve surgery within 198 hours	81 (15%)	7 (13%)	0.696

Data are depicted as number (percentage) or median (Q1-Q3)

CIED: cardiac implantable electronic devices

[a]included methicillin resistant staphylococci, penicillin resistant streptococci, and amoxicillin resistant enterococci

Supplementary Table 2. Multivariable analysis of predictors of persistent bacteraemia for at least 168 hours among patients with valve-associated infective endocarditis

	P	**aOR (95% CI)**
S. aureus	<0.001	10.24 (3.80-27.61)
Resistant bacteria[a]	<0.001	7.13 (2.53-20.22)
Sepsis	0.447	1.36 (0.62-3.00)
Acute native bone and joint infection	0.006	3.02 (1.38-6.62)
Thoracic embolic events	0.167	2.10 (0.73-3.26)
Tricuspid valve infective endocarditis	0.923	1.06 (0.34-5.99)
Source control		
Warranted; not performed within 168 hours	reference	reference
Warranted; performed within 168 hours	<0.001	0.25 (0.01-0.79)
Not warranted	<0.001	0.04 (0.01-0.10)

aOR: adjusted odds ratios, CI: confidence interval

[a]included methicillin resistant staphylococci, penicillin resistant streptococci, and amoxicillin resistant enterococci
